# Male Involvement in Maternal Health Care at Anomabo, Central Region, Ghana

**DOI:** 10.1155/2017/2929013

**Published:** 2017-11-21

**Authors:** Joshua Panyin Craymah, Robert Kwame Oppong, Derek Anamaale Tuoyire

**Affiliations:** ^1^Department of Internal Medicine and Therapeutics, School of Medical Sciences, University of Cape Coast, Cape Coast, Ghana; ^2^College of Distance Education, University of Cape Coast, Cape Coast, Ghana; ^3^Department of Community Medicine, School of Medical Sciences, University of Cape Coast, Cape Coast, Ghana

## Abstract

**Background:**

Globally, male involvement in maternal health care services remains a challenge to effective maternal health care accessibility and utilization.

**Objective:**

This study assessed male involvement in maternal health care services and associated factors in Anomabo in the Central Region of Ghana.

**Methods:**

Random sampling procedures were employed in selecting 100 adult male respondents whose partners were pregnant or had given birth within twelve months preceding the study. Pearson Chi-Square and Fisher's exact tests were conducted to assess the association of sociodemographic and enabling/disenabling factors with male involvement in maternal health care services.

**Results:**

Some 35%, 44%, and 20% of men accompanied their partners to antenatal care, delivery, and postnatal care services, respectively. Male involvement in antenatal care and delivery was influenced by sociodemographic (partner's education, type of marriage, living arrangements, and number of children) and enabling/disenabling (distance to health facility, attitude of health workers, prohibitive cultural norms, unfavourable health policies, and gender roles) factors.

**Conclusion:**

The low male involvement in maternal health care services warrants interventions to improve the situation. Public health interventions should focus on designing messages to diffuse existing sociocultural perceptions and health care provider attitudes which influence male involvement in maternal health care services.

## 1. Introduction

Maternal health care (MHC) service comprises services provided for women during pregnancy, delivery, and postnatal. Traditionally, maternal health issues have predominantly been seen and treated as a purely feminine matter [1]. Hence, men have traditionally been excluded from MHC services, thereby reinforcing the erroneous notion that pregnancy and the processes leading to childbirth are the preserve of women [2].

According to the recent global estimates by the World Health Organization (WHO), more than half a million women lose their lives from pregnancy-related complications worldwide every year, ninety-nine per cent (99%) of which occur in the less developed world [3]. In Sub-Saharan Africa, one out of every thirteen women dies of pregnancy-related causes compared with one in 4,085 women in industrialized countries [4]. For every maternal death, many more women suffer short-term injuries, infections, and disabilities during pregnancy or child birth each year.

The tendency to view maternal health as a woman's issue has contributed to a narrow focus of targeting mostly women, particularly mothers in intervention efforts. Most maternal and child health (MCH) programmes seek to address the health needs of women and children by engaging and educating pregnant women and mothers in care-seeking practices for themselves and their children. This has contributed to men being sidelined as far as reproductive health and MCH matters are concerned [2].

Male involvement in MHC has been described as a process of social and behavioural change that is needed for men to play more responsible roles in MHC with the purpose of ensuring women's and children's wellbeing [5]. Indeed, the value of direct male involvement in reducing maternal mortality cannot be overestimated. Referring to Millennium Development Goal 5 (MDG), an article in* Frontlines, *a monthly publication of the United States Agency for International Development (USAID) noted that “reducing maternal deaths by 75 percent throughout the world by 2015 will take the involvement of men in countries where it matters most” [6].

The involvement of men in maternal health arises from the numerous influences men have on almost all spheres of life [1, 7]. The poor attitude of men towards maternal health especially in Africa has been greatly attributed to the practice of male dominance, often called “patriarchy” [1]. Given the crucial role African men play in family decisions, their support and involvement in MHC are essential for healthy maternal and child welfare.

According to Green [8], social relationships determine people's ability to manage their sexual and reproductive health (SRH), with important implications not only for their health but also for other life choices. Some studies show that many negative conditions are avoidable if the pregnant woman gets social and psychological support, not only from high quality maternal and child health care but also from their social network, especially their partners [9, 10].

In spite of the important role of men in maternal health, studies exploring male involvement in MHC and factors that influence their participation are limited. The few available studies have to a large extent explored the perspective of women, but not men. One limitation of studying the subject based on women samples is that the perspectives of women could be a mere reflection of their feelings about the quality of their relationships with their male partners [11].

The level of male involvement in maternity care varies across communities and countries. There are various factors that could determine the level of male involvement. These could be sociodemographic, cultural, or even inherent factors in the health delivery systems [12–14]. The present study seeks to assess male involvement in MHC and associated factors at Anomabo in the Central Region of Ghana. Findings from the present study could inform the design of intervention strategies towards improving male involvement in MHC and MCH services in Anomabo and beyond.

## 2. Methods

### 2.1. Study Setting

The study was conducted in Anomabo, a town within the Mfantseman Municipality located along the Atlantic coastline of the Central Region of Ghana. According to the Ghana Statistical Service [15], Anomabo has a settlement population of 13,401 people consisting of 6,047 males and 7,354 females. There are 3,621 household and 1,579 houses. Majority of these houses are compound houses and semidetached houses [15]. The people of Anomabo are predominantly Fantes, with fishing, farming, and trading as their main economic activities. The Anomabo Health Centre is the only facility serving the health needs of the community.

### 2.2. Study Design and Sample Size Determination

This was a cross-sectional study involving male respondents whose partners were pregnant or had given birth within twelve months preceding the study. The sample size for the study was calculated from 3621 households in Anomabo Sub-district. The study employed Yamane [16] formula for sample size determination as indicated below:

(1) $$\begin{array}{c} Formula\;\;n=\frac{N}{1+N{\left(e\right)}^{2}}=\frac{3621}{1+3621{\left(0.1\right)}^{2}}=97.31, \end{array}$$

where *n* is sample size; *N* is the population size; *e* is the acceptable sampling error. 95% confidence level and *P* = 0.05 are assumed.

### 2.3. Sampling Procedure

Random sampling procedures were employed in selecting 100 adult (20 years or above) male respondents whose partners were pregnant or had given birth within twelve months preceding the study. To begin the process, a sample frame was constructed to include a list of all households (3621) in the community. The lottery method was then used to select household with eligible potential respondent from which consenting participants were selected and interviewed.

### 2.4. Data Collection Instrument

A questionnaire was designed for the study by the investigators (authors) and comprised of both close-ended and open-ended questions. The questionnaire was in three sections: Section A was designed to collect sociodemographic information; Section B sought to elicit information on the level of male involvement in maternal health care (antenatal care, delivery, and postnatal care); and Section C sought to collect information on enabling/disenabling factors influencing males' involvement in MHC.

Prior to data collection, the questionnaire was pretested at Biriwa, a close by community in the Mfantseman Municipality. Biriwa was chosen because it has similar sociodemographic and socioeconomic characteristics as the study area. This provided a means for ascertaining appropriateness of the questions for obtaining valid and reliable responses. All necessary adjustment and modifications were then made on the instrument before the actual data collection begun.

### 2.5. Data Processing and Analysis

The data collected from the field were edited for any inconsistencies and appropriately coded, after which the data was entered using Statistical Product and Service Solution (SPSS) software Version 21. Once entered, the data was exported to STATA Version 12.0 for cleaning and further analysis.

The dependent variable for the study was male involvement in specific MHC services: whether a respondent accompanied his pregnant partner to the health centre for antenatal care, delivery, and postnatal care. The independent variables considered in the study were grouped into two: sociodemographic (age, employment status, education of the man, education of the spouse, religion, type of marriage status, number of children, and living arrangement) and enabling/disenabling (distance to health facility, perception of MHC, poor spousal communication, prohibitive cultural norms, work schedules, gender roles, unfavourable health policies, financial problems, attitude of health workers, and long waiting time at the health facility) factors.

Both descriptive and inferential statistics were employed in the analyses. Pearson Chi-Square and Fishers exact tests were conducted to assess the bivariate association between the independent variables and dependent variable (whether a man accompanied his partner to antenatal care, delivery, and postnatal care). The bivariate analysis was also conducted to identify variables that show a significant relationship between independent variables and dependent variable. Significance level was set at *P* < 0.05.

### 2.6. Ethical Consideration

Ethical approval was obtained from the Institutional Review Board (IRB) of the University of Cape Coast (UCC). Additional approval was obtained from the Mfantseman Municipal Health Directorate (MMHD) before the study was conducted. Written informed consent was obtained from all participants after giving a description of the study. Confidentiality was seriously adhered to throughout the study processes.

## 3. Results

### 3.1. Sociodemographic Characteristics of Respondents

As indicated in Table 1, the mean age of the 100 respondents sampled for this study was 39.7 (SD 10.2) years with most (36%) between the ages of 30–39 years of age. More than half (55%) of the respondents had elementary/JSS education, while about two-thirds (59%) reported that their partners had no education. Most respondents were in monogamous unions (90%), unemployed (72%), and of Christian (65%) faith. In terms of parity, 45% of the respondents had less than three children.

### 3.2. Enabling/Disenabling Factors in Maternal Health Care

Table 2 presents a summary of possible enabling/disenabling factors of MHC reported by respondents in the study. The results show that 55% of the respondents lived together with their partners. Eight out of every ten (80%) respondents lived less than five kilometres away from the health centre, while 61% perceived the MHC services offered at the health centre to be easily accessible. In addition, most respondents indicated that poor spousal communication (93%), prohibitive cultural norms (69%), work schedules (79%), unfavourable health policies (85%), financial problem (53%), attitudes of health workers (90%), long waiting time at health facility (83%), and gender roles of men (50%) affected their involvement in MHC.

### 3.3. Male Involvement in MHC Services

**Figure 1:**
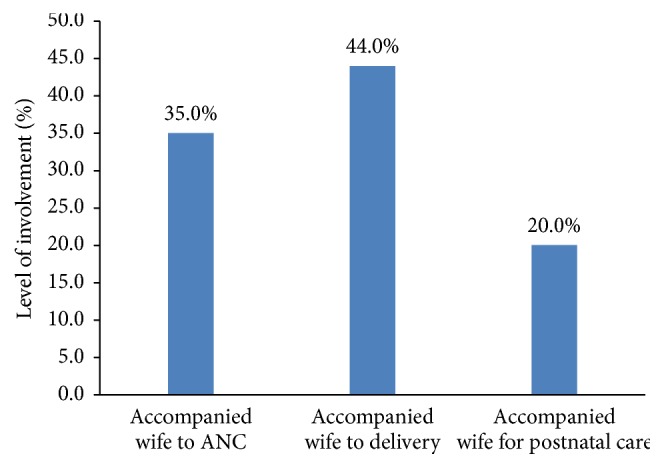
Level of male involvement in maternal health care (*N* = 100). Source: Fieldwork, 2013.

The results on male involvement in various MCH services show that 35% of respondents accompanied their partners to antenatal care during pregnancy, while 44% accompanied their partners to delivery. One-fifth (20%) of the respondents accompanied their partners for postnatal care services (Figure 1).

### 3.4. Factors Associated with Male Involvement in MHC

#### 3.4.1. Sociodemographic Factors

Table 3 shows results of bivariate association between sociodemographic factors and male involvement in each of the MHC services (antenatal care, delivery, and postnatal care) considered in the study. Male involvement in antenatal care and delivery was significantly (*P* < 0.05) associated with partner's education, type of marriage, living arrangement, and number of children. In contrast, no significant association was found between any of the sociodemographic factors and male involvement in postnatal care. As in Table 3, male involvement in MHC was significantly higher among respondents whose partners had tertiary education (antenatal: 100%, delivery: 100%) than those with no education (antenatal: 0%, delivery: 0%).

Similarly, male involvement in MHC was significantly higher among respondents in monogamous marriages (antenatal: 39%, delivery: 49%) than those in polygamous marriages (antenatal: 0%, delivery: 0%). Male involvement in antenatal care was significantly (*P* = 0.020) higher among respondents who were living together (45%) with their partners than their counterparts who were not living together (22%) with their partners. In terms of number of children, male involvement in MHC was significantly higher among respondents with one to three children (antenatal: 51%, delivery: 58%) than those with no children (antenatal: 14%, delivery: 43%).

#### 3.4.2. Enabling/Disenabling Factors

As in the case of sociodemographic factors, no significant association was found between any of the enabling/disenabling factors and male involvement in postnatal care.  Table 4 shows that distance to health facility (less than 5 km: 40% versus more than 5 km: 15%) and attitude of health workers (yes: 39% versus no: 0%) were the enabling/disenabling factors significantly (*P* < 0.05) associated with male involvement in antenatal. On the other hand, prohibitive cultural norms, unfavourable health policies, and gender roles were the enabling/disenabling factors found to be significantly (*P* < 0.05) associated with male involvement in both antenatal and delivery.

## 4. Discussion

The study revealed low involvement of men in MHC, with variations in the proportion of men accompanying their partners to antenatal care, delivery, and postnatal care services. About 35% accompanied their partners to antenatal care, 44% to delivery, and 20% for postnatal care. A previous study conducted by Tweheyo et al. [17] in Northern Uganda reported that 48% of men accompanied their partners during delivery but 65% did the same for antenatal care. The low involvement of men in antenatal, delivery, and postnatal care found in the current study could be attributed to a number of factors which have been highlighted in the results, as well as extant literature.

Various researchers have pointed to the fact that a partner's education level and employment status could have an influence on the level of male involvement in maternal health services [18, 19]. Thus, high levels of education among pregnant women are associated with high levels of involvement of their male partners in MHC. Our analysis showed that the partners of most respondents had no education (59%), and this was found to be significantly associated with male involvement in MHC services, particularly antenatal care and delivery. This suggests that perhaps uneducated women are less likely to discuss and involve men in decisions on maternal health issues than their more educated counterparts [20], which could explain the low level of involvement of males in antenatal, delivery, and postnatal services found in this study. The high level of unemployment (72%) could also be an indicator of financial inaccessibility to health facility, thereby contributing to low male involvement in MHC.

It was revealed in this study that prohibitive cultural norms and gender roles play a role in male involvement in MHC. Men often see pregnancy and maternal health related issues as women's responsibility. For instance, Mullick et al. [21] indicated that men hold on to their cultural beliefs that a man may lose “strength” if he is present during the birth of his baby and therefore men do not escort the women for maternal health services. Similar views have been reported in Kenya [22, 23].

From our study, there was a significant relationship between unfavourable health policies and male involvement in MHC. Green [8] noted that researchers and reproductive health service providers have tended to describe women's disadvantaged positions without men's roles. This has resulted in the case where most reproductive health programs designed to improve women reproductive health consider men as part of the problem and not part of the solution. Such health policies addressing maternal health issues focus primarily on women and children than men. This demoralizes the intention of men to accompany their partners to assess MHC. The failure to incorporate men in maternal health promotion, prevention, and care programs by policy makers, program planners, and implementers has had a serious impact on male involvement in the health of women including MHC [8, 24, 25].

Lastly, the attitudes of health workers at the health facility accounted for low male involvement in MHC in our study. The study is consistent with a study conducted by Byamugisha et al. [26]. They reported that harsh and critical language directed at Ugandan women from skilled health professionals was a barrier to male participation. Harsh treatment of men by health providers discouraged them from returning or participating in prevention of mother-to-child transmission (PMTCT) of HIV activities. In Turkey, it was observed that health care workers were not supporting men who wanted to join in MHC services, and as such a lot of men who visited the clinic with their wives had to stop at the door of the clinic [27]. Thus, low male involvement in MHC could result from the fear of men being the subject of verbal, emotional, and sometimes physical abuse [28].

## 5. Conclusion

Male involvement MHC in Anomabo in the Central Region of Ghana is low. Various sociodemographic (partner's education, type of marriage, and number of children) and enabling/disenabling (distance to health facility, attitude of health workers, prohibitive cultural norms, unfavourable health policies, and gender roles) factors are associated with male involvement in MHC services.

There is the need for urgent interventions to scale up the involvement of men in MHC utilization. Public health interventions should focus on designing messages bearing in mind the variety of sociodemographic and enabling/disenabling factors outlined in this study. Specifically, improving access to formal education could help diffuse existing sociocultural perceptions of men accompanying their partners to antenatal care, delivery, and postnatal care, while encouraging positive health care provider attitudes towards male involvement in MHC services.

## Figures and Tables

**Table 1:** Sociodemographic characteristics of male respondent (*N* = 100).

Characteristics	Frequency (*N*)	Percentage (%)
Age	Mean = 39.7 (±10.2)	
20–29	17	17.0%
30–39	36	36.0%
40–49	25	25.0%
50–59	22	22.0%
Education of Man		
None	19	19.0%
Elementary/JHS	55	55.0%
Secondary/technical	20	6.0%
Tertiary	6	20.0%
Partners education		
None	59	59.0%
Elementary/JHS	24	24.0%
Secondary/technical	10	10.0%
Tertiary	7	7.0%
Marriage relationship		
Monogamous	90	90.0%
Polygamous	10	10.0%
Employment status		
Employed	28	28.0%
Not employed	72	72.0%
Religion		
Christian	65	65.0%
Muslim	25	25.0%
Traditional	10	10.0%
Number of children		
No child yet	21	21.0%
Less than 3	45	45.0%
More than 3	34	34.0%

Source: Fieldwork, 2013.

**Table 2:** Enabling/disenabling factors of male involvement in maternal health care (*N* = 100).

Characteristics	Frequency (*N*)	Percentage (%)
Couple living together		
Yes	55	55.0%
No	45	45.0%
Distance to H/F		
Less than 5 km	80	80.0%
More than 5 km	20	20.0%
Perception of MHC		
Easily accessible	61	61.0%
Not accessible	39	39.0%
Poor spousal communication		
Yes	93	93.0%
No	7	7.0%
Prohibitive cultural norms		
Yes	69	69.0%
No	31	31.0%
Work schedules of men		
Yes	79	79.0%
No	21	21.0%
Unfavourable health policies		
Yes	85	85.0%
No	15	15.0%
Financial problems		
Yes	53	53.0%
No	47	47.0%
Attitudes of health workers		
Yes	90	90.0%
No	10	10.0%
Long waiting time at H/F		
Yes	83	83.0%
No	17	17.0%
Gender roles		
Yes	50	50.0%
No	50	50.0%

Source: Fieldwork, 2013; MHC: maternal health care; H/F: health facility.

**Table 3:** Sociodemographic factors associated with male involvement in antenatal care, delivery, and postnatal care (*N* = 100).

Variable	Antenatal care			Delivery			Postnatal care		
	Yes	No	Total	Yes	No	Total	Yes	No	Total
Age									
20–29	3 (17.6)	14 (82.4)	17	9 (52.9)	8 (47.1)	17	4 (23.5)	13 (76.5)	17
30–39	11 (30.6)	25 (69.4)	36	13 (36.1)	23 (63.9)	36	7 (19.4)	29 (80.6)	36
40–49	13 (52)	12 (48)	25	14 (56)	11 (44)	25	5 (20)	20 (80)	25
50–59	8 (36.4)	14 (63.3)	22	8 (36.4)	14 (63.6)	22	4 (18.2)	18 (18)	22
*P* value	0.12			0.33			0.98		
Education of man									
None	4 (21.1)	15 (78.9)	19	6 (31.6)	13 (68.4)	19	3 (15.8)	16 (84.2)	19
Elementary/JHS	23 (41.8)	32 (58.2)	55	27 (49.1)	28 (50.9)	55	12 (21.8)	43 (78.2)	55
Secondary/technical	0 (0.0)	6 (100)	6	11 (55)	9 (45)	20	4 (20)	16 (80)	20
Tertiary	8 (40)	12 (60)	20	0 (0)	6 (100)	6	1 (16.7)	5 (83.3)	6
*P* value	0.10			0.06			0.37		
Partners education									
None	14 (23.7)	45 (76.3)	59	23 (39.0)	36 (61.0)	59	14 (23.7)	45 (76.3)	59
Elementary/JHS	12 (50.0)	12 (50.0)	24	12 (50)	12 (50.0)	24	5 (20.8)	19 (79.2)	24
Secondary/technical	2 (20.0)	8 (80.0)	10	2 (20)	8 (80.0)	10	1 (10.0)	9 (90.0)	10
Tertiary	7 (100)	0 (0.0)	7	7 (100)	0 (0.0)	7	0 (0.0)	7 (100)	7
*P* value	**0.000**			**0.01**			0.41		
Type of marriage									
Monogamous	35 (38.9)	55 (61.1)	90	44 (48.9)	46 (51.1)	90	19 (21.1)	71 (78.9)	90
Polygamous	0 (0.0)	10 (100)	10	0 (0)	10 (100)	10	1 (10)	9 (90)	10
*P* value	**0.01**			**0.000**			0.68		
Employment status									
Formal	7 (25)	21 (75)	28	11 (39.3)	17 (60.7)	28	6 (21.4)	22 (78.6)	28
Informal	28 (38.9)	44 (61.1)	72	33 (45.8)	39 (54.2)	72	14 (19.4)	58 (80.6)	72
*P* value	0.19			0.55			0.82		
Religion									
Christian	21 (32.3)	44 (67.7)	65	28 (43.1)	37 (56.9)	65	12 (18.5)	53 (81.5)	65
Muslim	8 (32.0)	17 (68.0)	25	10 (40.0)	15 (60.0)	25	7 (28.0)	18 (72.0)	25
Traditional	6 (60.0)	4 (40.0)	10	6 (60.0)	4 (40.0)	10	1 (10.0)	9 (90.0)	10
*P* value	0.26			0.53			0.43		
Number of children									
No child yet	3 (14.3)	18 (85.7)	21	9 (42.9)	12 (57.1)	21	5 (23.8)	16 (76.2)	21
Less than 3	23 (51.1)	22 (48.9)	45	26 (57.8)	19 (42.2)	45	8 (17.8)	37 (82.2)	45
More than 3	9 (26.5)	25 (73.5)	34	44 (44.0)	56 (56.0)	34	7 (20.6)	27 (79.4)	34
*P* value	**0.01**			**0.02**			0.85		

*P* value < 0.05 in bold.

**Table 4:** Enabling/disenabling factors associated with male involvement in antenatal care, delivery, and postnatal care (*N* = 100).

Variable	Antenatal care			Delivery			Postnatal care		
	Yes	No	Total	Yes	No	Total	Yes	No	Total
Couple living together									
Yes	25 (45.5)	30 (54.5)	55	26 (47.3)	29 (52.7)	55	10 (18.2)	45 (81.8)	55
No	10 (22.2)	35 (77.8)	45	18 (40)	27 (60)	45	10 (22.2)	35 (77.8)	45
*P* value	**0.02**			0.47			0.62		
Distance to H/F									
Less than 5 km	32 (40)	48 (60)	80	37 (46.2)	43 (53.8)	80	15 (18.8)	65 (81.2)	80
More than 5 km	3 (15)	17 (85)	20	7 (35)	13 (65)	20	5 (25)	15 (75)	20
*P* value	**0.04**			0.37			0.54		
Perception of MHC									
Easily accessible	23 (37.7)	38 (62.3)	61	26 (42.6)	35 (57.4)	61	12 (19.7)	49 (80.3)	61
Not accessible	12 (30.8)	27 (69.2)	39	18 (46.2)	21 (53.8)	39	8 (20.5)	31 (79.5)	39
*P* value	0.48			0.73			0.92		
Poor spousal communication									
Yes	35 (37.6)	58 (62.4)	93	42 (45.2)	51 (54.8)	93	18 (19.4)	75 (80.6)	93
No	0 (0)	7 (100)	7	2 (28.6)	5 (71.4)	7	2 (28.6)	5 (71.4)	7
*P* value	0.09			0.46			0.63		
Prohibitive cultural norms									
Yes	15 (21.7)	54 (78.3)	69	23 (33.3)	46 (66.7)	67	12 (17.4)	57 (82.6)	69
No	20 (64.5)	11 (35.5)	31	21 (67.7)	10 (32.3)	31	8 (25.8)	23 (74.2)	31
*P* value	**0.000**			**0.000**			0.33		
Work schedules of men									
Yes	25 (31.6)	54 (68.4)	79	32 (40.5)	47 (59.5)	79	15 (19.0)	64 (81.0)	79
No	10 (47.6)	11 (52.4)	21	12 (57.1)	9 (42.9)	21	5 (23.8)	16 (76.2)	21
*P* value	0.17			0.17			0.76		
Unfavourable health policies									
Yes	34 (40.0)	51 (60.0)	85	42 (49.4)	43 (50.6)	85	17 (20.0)	68 (80.0)	85
No	1 (6.7)	14 (93.3)	15	2 (13.3)	13 (86.7)	15	3 (20.0)	12 (80.0)	15
*P* value	**0.01**			**0.01**			1.00		
Financial problems									
Yes	16 (34.0)	31 (66.0)	47	21 (44.7)	26 (55.3)	47	10 (21.3)	37 (78.7)	47
No	19 (35.8)	34 (64.2)	53	23 (43.4)	30 (56.6)	53	10 (18.9)	43 (81.1)	53
*P* value	0.85			0.89			0.76		
Attitudes of health workers									
Yes	35 (38.9)	55 (61.1)	90	42 (46.7)	48 (53.3)	87	17 (18.9)	73 (81.1)	90
No	0 (0.0)	10 (100)	10	2 (20.0)	8 (80.0)	10	3 (30.0)	7 (70.0)	10
*P* value	**0.01**			0.12			0.41		
Long waiting time at H/F									
Yes	27 (32.5)	56 (67.5)	83	35 (42.2)	48 (57.8)	83	17 (20.5)	66 (79.5)	83
No	8 (47.1)	9 (52.9)	17	9 (52.9)	8 (47.1)	17	3 (17.6)	14 (82.4)	17
*P* value	0.25			0.42			1.00		
Gender roles									
Yes	23 (46.0)	27 (54.0)	50	29 (58.0)	21 (42.0)	50	12 (24.0)	38 (76.0)	50
No	12 (24.0)	38 (76.0)	50	15 (30.0)	35 (60.0)	50	8 (16.0)	42 (84.0)	50
*P* value	**0.02**			**0.01**			0.32		

*P* value < 0.05 in bold. H/F: health facility; MHC: maternal health care.
